# The Scale of Positive and Negative Experience (SPANE): Psychometric Properties and Normative Data in a Large Chinese Sample

**DOI:** 10.1371/journal.pone.0061137

**Published:** 2013-04-03

**Authors:** Feng Li, Xinwen Bai, Yong Wang

**Affiliations:** 1 Key Laboratory of Mental Health, Institute of Psychology, Chinese Academy of Sciences, Beijing, China; 2 Key Laboratory of Behavioral Science, Institute of Psychology, Chinese Academy of Sciences, Beijing, China; George Mason University/Krasnow Institute for Advanced Study, United States of America

## Abstract

**Background:**

The Scale of Positive and Negative Experience (SPANE) is a new instrument that assesses subjective feelings of well-being and ill-being and overcome several limitations of previous popular instruments. The current study examined the scale's psychometric properties with a large Chinese sample.

**Principal Findings:**

Data were collected form 21,322 full-time workers from the power industry. The psychometric properties were assessed in term of internal consistency reliability, factorial validity, convergent validity, and measurement invariance across gender, age, marital status, education level, and income level. The results demonstrate that the SPANE has high internal consistency reliability, a correlated two-factor structure (with the uniqueness of three general and specific items of positive and negative feelings allowed to correlate with each other), strict equivalence across gender, age and marital status, and strong equivalence across education and income. Furthermore, the SPANE converges well with two measures of life satisfaction.

**Conclusion:**

The Chinese version of the SPANE behaves consistently with the original and can be used in future studies of emotional well-being. The scale norms are presented in terms of percentile rankings, and implications and directions for future research are discussed.

## Introduction

Subjective well-being (SWB), a construct that reflects people's subjective and global evaluations of their lives as well as positive and negative affective reactions, has attracted tremendous interest from psychologists, sociologists and economists [1]. Over the past decades, researchers have put considerable effort into defining and measuring SWB, discovering predictive variables, comparing the levels of SWB across different countries, and developing strategies to promote and maintain individual SWB [2]–[5]. Consequently, many researchers have developed scales of SWB and its related constructs. For instance, Diener and his colleagues (2010) created a new measure called the Scale of Positive and Negative Experience (SPANE) to assess the affective component of SWB [6].

The affective component of SWB reflects the balance in a person's life between pleasant affect and unpleasant affect. In a series of studies, Bradburn (1969) found that (a) the correlations between positive and negative items were very low; (b) the correlations between items within positive or negative affect scales were much higher; and (c) positive and negative affect correlated differently with several external variables, such as anxiety and social participation [7]. These findings established the foundation for the distinction between positive affect (PA) and negative affect (NA) as two separate components of SWB [3], [8]. A number of researchers subsequently provided support for the distinction between PA and NA with different correlations. For instance, Diener and Emmons (1985) found that all the results of factor analysis, inter-item correlations, correlations among affective means, and correlations with external variables supported the relative independence of positive and negative affect in people's lives, although pleasant and unpleasant affect states did vary inversely only over short time spans [9]. Moreover, based on the analysis of a number of studies of self-reported mood, Watson and Tellegen (1985) presented a basic, consensual model of positive and negative affect [10]. These two factors have been used as dependent, independent, or control variables in numerous studies and in disciplines outside of psychology from business to politics [11]. Despite this evidence, the separation of pleasant and unpleasant emotions remains a contentious issue in terms of the time frame [12], response styles [13], response format [14], and arousal of item content [15]. However, in a recent review, Schimmack (2008) concluded that the independence of PA and NA is broadly supported by empirical research. However, the two factors may not be strictly independent or orthogonal (r = .00), and PA and NA have different predictors and may even co-occur at the same moment. Thus, it seems necessary to separately assess positive and negative affect as a way to fully understand SWB [8].

Given the two-factor structure of affect, numerous scales have been developed to assess pleasant and unpleasant emotions in a variety of research areas, such as Affect Balance Scale (ABS) [7], Positive and Negative Affect Schedule (PANAS) [16], and Hedonic Balance Scale (HBS) [17]. Of these scales, the most widely used is the PANAS [6], which has been particularly well validated and cited in more than 2,000 scholarly papers in various cultural contexts [11]. However, several limitations exist for this measure. First, the PANAS may not reflect feelings of enhanced well-being because it includes some items that are not considered feelings (e.g., “strong”, “alert”, “active”, and “determined”), and it omits some core emotional feelings (e.g., “bad”, “joy”) [6]. Second, the PANAS does not consider the difference in the desirability of feelings in different contexts or cultures (e.g., Hong Kong Chinese desire low-arousal positive affect more than do European Americans [6], [18]. Third, there is considerable redundancy in the PANAS items because models with items from the same original word pool co-clusters correlated best with the data [11], [19]. To overcome some of the shortcomings of the PANAS, Diener and his colleagues (2010) developed the new Scale of Positive and Negative Experience (SPANE) to assess a broad range of pleasant and unpleasant feelings by asking people to report their feelings in terms of their duration after recalling their activities and experiences during the previous 4 weeks [6].

The SPANE consists of 12 items: six items assess positive feelings, and the other six assess negative feelings. For both positive and negative feelings, three items are general (e.g., positive, negative) and three are specific (e.g., happy, sad). The broad descriptors allow the SPANE to reflect the full range of people's desirable and undesirable experiences without creating an exhaustive word list. Furthermore, the SPANE can capture positive and negative feelings regardless of their sources, arousal level or cultural context. The specific words reflect the most important forms of feelings related to well-being and ill-being and capture feelings from around the emotion circumplex. The time frame of four weeks provides a balance between sampling adequacy of feelings and memory accuracy. Furthermore, the use of the time response style should decrease the ambiguity of people's understanding of the scale and should enhance the validity of the SPANE [6].

The new scale showed good psychometric properties in the original research [6] and one follow-up validation study in Portugal [5], with an internal consistency coefficient (Cronbach's alpha) from .81 to .90. The separate results of the principal axis factor analysis for the positive (SPANE-P) and negative (SPANE-N) items indicated that both the SPANE-P and the SPANE-N produced one strong factor with an eigenvalue above one and factor loadings from .49 to .81, accounting for 61 and 53%% of the variance in the scale, respectively [6]. Another result of the principal component analysis for both positive and negative items showed that the SPANE-P and the SPANE-N were separately loaded onto two strong factors with eigenvalues greater than one and factor loadings from .66 to .85, accounting for 62% of the variance [5]. Moreover, the multi-group confirmatory factor analysis confirmed that this two-factor model was invariant across full-time worker and student samples [5]. In these two studies, the scale performed well in terms of convergent validity with other measures of emotion, happiness, and life satisfaction. Furthermore, the SPANE-P and SPANE-N correlated significantly each other.

Although the psychometric characteristics of the SPANE are encouraging, more work is needed, especially on broader populations and the convergence of cultures and groups [6]. In response to this need, the present study aimed to evaluate the psychometric properties of the SPANE in terms of item analysis, internal consistency reliability, factorial validity and measurement invariance with a large Chinese sample. Although Silva and Caetano (2013) assessed the invariance of the SPANE across full-time worker and student samples [5], the present study addresses the issue of measurement invariance more thoroughly, especially concerning invariance across gender, age, educational level, and income. Also, with increasing interest in Chinese SWB research in recent years [2], [20]–[25], we believe that the present study will establish a foundation for the further use of the SPANE in the understanding of Chinese people's emotional wellbeing.

## Materials and Methods

### Ethics Statement

This research protocol was approved by the Ethics Committee of the Institute of Psychology, CAS and competent department of the power industry. All participants were promised to be anonymous and confidential in the data analysis.

### Procedure and Participants

All of the data used in the current research are part of a large-scale survey that aimed to investigate views of happiness among full-time employees of the power industry. The survey was conducted in October 2011 in 176 enterprise units located in Guangdong, Guangxi, Guizhou, Yunnan and Hainan provinces. A sampling plan was designed according to location, unit size, gender, age, and position. The participants were invited to complete the online questionnaire by email.

Of the 21,716 employees who were invited, 21,322 respondents provided complete data on the items of the SPANE, yielding a valid response rate of 98.2%. Their ages ranged from 18 to 60, and 67.1% were male. Most of the respondents were married with a junior college to college education level. Detailed demographic information on the 21,322 respondents as well as scores on the SPANE for each demographic group is summarized in Table 1.

**Table 1:** Participants' demographic data and SPANE scores (*N = 21,322*).

Variables		Frequency (%)	Mean (SD) of the SPANE score		
			SPANE-P	SPANE-N	SPANE-B
Gender	Male	14314 (67.1%)	21.04 (4.67)	14.21 (4.86)	6.83 (8.21)
	Female	7008 (32.9%)	21.02 (4.56)	14.69 (4.79)	6.33 (8.11)
Age	18–25	3084 (14.5%)	21.18 (4.66)	14.29 (4.72)	6.89 (8.12)
	26–30	4165 (19.5%)	20.77 (4.58)	14.84 (4.72)	5.93 (8.09)
	31–35	4089 (19.2%)	20.71 (4.66)	14.62 (4.78)	6.09 (8.24)
	36–40	3557 (16.7%)	21.06 (4.62)	14.31 (4.87)	6.75 (8.15)
	41–45	2696 (12.6%)	21.31 (4.58)	13.88 (4.90)	7.43 (8.17)
	46–50	1720 (8.1%)	21.62 (4.74)	13.67 (4.99)	7.94 (8.25)
	51–55	1049 (4.9%)	21.20 (4.46)	14.26 (5.01)	6.94 (7.88)
	56–60	962 (4.5%)	20.96 (4.68)	14.42 (5.03)	6.54 (8.39)
Education level	Junior middle school and below	262 (1.2%)	21.15 (5.09)	13.84 (5.21)	7.31 (7.54)
	High school	2742 (12.9%)	20.69 (4.72)	14.56 (5.07)	6.14 (8.22)
	Junior College	7811 (36.6%)	20.90 (4.70)	14.44 (4.95)	6.45 (8.29)
	College	9486 (44.5%)	21.15 (4.54)	14.30 (4.69)	6.85 (8.08)
	Master and above	1021 (4.8%)	21.83 (4.49)	14.03 (4.66)	7.80 (8.19)
Personal income per month (RMB)	1001–2000	5202 (24.4%)	20.08 (4.87)	15.06 (4.99)	5.02 (8.38)
	2001–5000	11358 (53.3%)	21.14 (4.56)	14.28 (4.76)	6.86 (8.10)
	5001–10000	3734 (17.5%)	21.78 (4.35)	13.88 (4.71)	7.90 (7.85)
	10001–20000	736 (3.5%)	21.96 (4.39)	13.73 (5.03)	8.23 (7.96)
	More than 20000	292 (1.4%)	22.11 (4.26)	13.47 (4.92)	8.65 (7.87)
Marital status	Never married	5266 (24.7%)	20.91 (4.67)	14.62 (4.75)	6.29 (8.21)
	Married/With Spouses Separated	2378 (11.2%)	20.84 (4.65)	14.60(4.99)	6.24 (8.18)
	Married	13028 (61.1%)	21.17 (4.60)	14.20 (4.85)	6.98 (8.17)
	Divorced	394 (1.8%)	19.85 (4.40)	14.89 (4.70)	4.96 (7.37)
	Widow	41 (0.2%)	19.95 (5.06)	14.73 (4.57)	5.22 (7.34)
	Others	215 (1.0%)	19.97 (5.16)	15.02 (4.75)	4.95 (8.57)

### Materials

#### The Scale for Positive and Negative Experience (SPANE)

[6] This measure consists of two six-item subscales assessing people's positive and negative experiences over the previous 4 weeks. The answers are given on a five-point scale ranging from 1 (very rarely or never) to 5 (very often or always). Due to the partial independence of the two types of feelings, the positive and negative scales are scored separately. Both the summed positive (SPANE-P) score and the negative (SPANE-N) score can range from 6 to 30. These two scores can be combined by subtracting the negative score from the positive one, resulting in the balance (SPANE-B) score with a range from −24 to 24.

#### Satisfaction with Life Scale (SWLS)

[9] This scale is a five-item measure assessing satisfaction with a person's life as a whole. One example of the items is “the conditions of my life are excellent”. Responses are given on a seven-point scale with a range from 1 (strongly disagree) to 7 (strongly agree).

*Satisfaction with Life as a Whole* [25] This single item asks about the participant's overall satisfaction with his/her life in terms of his/her own life and personal circumstances. It is answered on an 11-point scale from 0 (completely dissatisfied) to 10 (completely satisfied).

### Analysis strategies

Item analysis was conducted using SPSS Version 20 to examine item and scale properties, such as the mean, standard deviation, skewness, kurtosis, corrected item-total score correlations, and internal consistency (Cronbach's α). Confirmatory factor analysis (CFA) and multi-group CFA were performed using Amos version 20 with the maximum likelihood method to further examine the factorial validity and measurement invariance across gender, age, education and income. Furthermore, an independent t-test and Pearson's correlations were calculated to examine the effects of descriptive variables on the scale scores and the convergent validity with other measures of well-being.

In the CFA analysis, as is standard practice to examine factorial validity, the first model to be evaluated was the single-factor model with all 12 items loaded. Then, the fit of the two-factor model with correlated positive experiences and negative experiences was tested. The model evaluation relied on the comparative fit index (CFI), the root mean square error of approximation (RMSEA), -the standardized root mean squared residual (SRMR), and Akaike's information criterion (AIC). According to Hu and Bentler (1999), when identifying a relatively good fit between the hypothesized model and the data, the stringent cutoff for the CFI should be greater than or equal to 0.95, less than or equal to 0.05 for the RMSEA, and less than 0.08 for the SRMR [26]. In addition, when comparing non-nested models, AIC could be compared where the smaller the better.

In the measurement invariance analysis, as the baseline, the model without invariance constraints was evaluated to determine whether patterns of factor structures in different groups were the same (configural invariance). After configural invariance was established, further factor loadings, structural variances and intercepts, and item uniqueness were systematically constrained to equal across the groups to test the satisfaction of weak invariance, strong invariance, and strict invariance models. Then, the model fits of those constrained models were compared to that of the configural model to determine whether the equality constraints resulted in a worse fit. The difference in χ^2^ between two nested models (Δχ^2^) is commonly used during such process. However, Δχ^2^ can be misleading because it is highly sensitive to sample size [27], [28]. Due to the large sample size in the current study, the changes in CFI and McDonald's (1989) noncentrality index (Mc) could be used to establish measurement invariance because they have been proven to be much less sensitive to sample size and more sensitive to a lack of invariance than Δχ^2^ [28].

## Results

### Item Analysis and Consistency Reliability of the SPANE

Table 2 presents the results of the item and reliability analysis for the SPANE. The corrected item-total correlations of each item score with its subscale score were in the range of .66 to .83, and all were higher than the traditional cutoff value of .30 (p.184) [29]. The range of skewness (.11 to−.67) and kurtosis (−.11 to .58) values indicated that the distribution was normal, and the current data were appropriate for the application of confirmatory factor analyses with the maximum likelihood method [2], [30].

**Table 2:** Results of item analysis for the SPANE (*N = 21,322*).

Items	Mean	SD	Skewness	Kurtosis	Commonalities	Corrected Item-Total Correlation	Cronbach's Alpha if Item Deleted
*Positive feelings*							
Positive	3.74	0.89	−0.67	0.58	0.48	0.66	0.92
Good	3.62	0.87	−0.50	0.39	0.64	0.76	0.90
Pleasant	3.54	0.90	−0.41	0.24	0.77	0.83	0.89
Joy	3.46	0.95	−0.35	0.04	0.76	0.83	0.89
Happy	3.42	0.91	−0.22	0.10	0.69	0.79	0.90
Contented	3.25	0.98	−0.22	−0.11	0.60	0.74	0.91
*Negative feelings*							
Negative	2.35	0.97	0.21	−0.42	0.57	0.71	0.89
Bad	2.39	0.96	0.18	−0.35	0.65	0.76	0.89
Unpleasant	2.53	0.97	0.11	−0.22	0.70	0.78	0.88
Sad	2.30	0.99	0.28	−0.42	0.70	0.79	0.88
Afraid	2.31	1.01	0.31	−0.49	0.52	0.69	0.90
Angry	2.48	0.96	0.14	−0.27	0.58	0.72	0.89

### Factorial Validity Analysis

As shown in Table 3, the two-factor model (model 2a) with positive and negative experiences loaded on two correlated factors fit the current data better than the unidimensional structure (model 1a) of the SPANE (Δχ^2^ = 48809.3, Δdf = 1, p<.001). However, this two-factor model could not be supported with a CFA value less than .95 and a RMSEA value more than .10. The residual covariance revealed that there were strong relationships among general feelings and specific feelings of the positive and negative experience items, such as good, positive, and pleasant and sad, angry, and afraid.

**Table 3:** Fit indices for CFA models of the SPANE (best fitting model in bold).

Models	χ^2^	Df	CFI	SRMR	AIC	RMSEA
1a. Single factor	64632.7	54	.645	.1545	64680.70	.237
1b. Single factor, correlated errors (CE) permitted	19636.5	42	.892	.1318	19708.53	.148
2a. Positive experience (PE) and negative experience (NE) as correlated factors	15823.4	53	.913	.0503	15873.43	.118
**2b. PE and NE as correlated factors, CE permitted**	**4611.2**	**41**	**.975**	**.0283**	**4685.23**	**.072**
3. Bifactor model, PE and NE as correlated factors	6707.3	41	.963	.0309	6781.29	.087

Note. Values >.90 for the CFI indicate a reasonable fit, whereas those >.95 suggests a good fit. Values <.05 for the RMSEA indicate a good fit, and values between .05 and .08 for the RMSEA indicate a reasonable fit. Values ≤.08 for the SRMR indicate a good fit; for AIC, the smaller the better.

**Figure 1:**
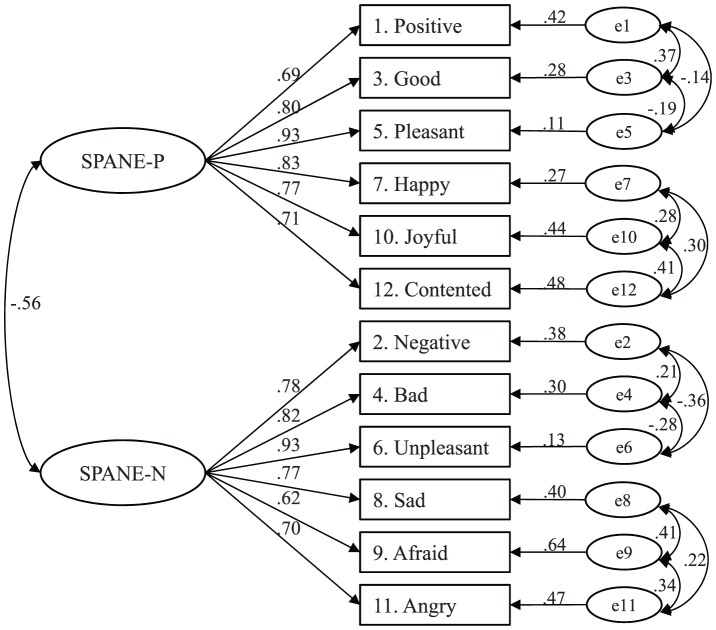
Graphical representation of the correlated two-factor model of the SPANE (model 2b); the factor loadings are standardized loadings.

To account for this phenomenon, three models were tested. First, on the basis of the same two-factor model, the uniqueness of three general and specific items of positive and negative feelings were allowed to be correlated with each other, as shown in Figure 1 (model 2b). This revised two-factor model assumed that all six positive and negative items assessed the same meanings of positive and negative feelings, respectively, but the three general items and specific items shared distinct meanings due to their different categories of feelings. The results in table 2 indicate that this revised two-factor model was acceptable (χ^2^_(41)_ = 4611.2; CFI = .975; SRMR = .0283; RMSEA = .072). Figure 1 presents a schematic representation of the standardized solution for this revised two-factor model. All the standardized factor loadings of the SPANE were above .50 and significant at the .01 level. The correlation between positive feelings and negative feelings was −.56 with p<.01.

Second, we specified a revised one-factor model (model 1b) with the same correlated uniqueness to the revised two-factor model (model 2b). The CFA results indicated that this revised one-factor model was not supported (χ^2^_(42)_ = 19636.5; CFI = .892; SRMR = .1318; RMSEA = .148), although it fit the data better than the original one-factor model (model 1a; Δχ^2^ = 44996.2, Δdf = 12, p<.001). Obviously, the revised two-factor model (model 2b) had a significantly better fit (Δχ^2^ = 15025.3, Δdf = 1, p<.001) than the revised unidimensional structure.

In recent research on the structure of the PANAS, Leue and Beauducel (2011) found that the best model fit occurred for the bifactor model, which includes a factor for positive affect and negative affect as well as an additional general factor [31]. We also specified a bifactor model of the SPANE (model 3) with a general factor accounting for the variance of the items that are not related to SPANE-P and SPANE-N factors. The CFA results showed that the bifactor model was acceptable (χ^2^_(41)_ = 6707.3; CFI = .963; SRMR = .0309; RMSEA = .087) but fit the data worse than the revised two-factor model with bigger AIC (ΔAIC = 2096.06).

Taken together, the revised two-factor model with correlated errors provided a superior model fit and could be used as the baseline model for the following invariance test. This finding means that positive and negative feelings were statistically separable into two strongly inversely correlated factors even when measurement error was controlled.

### Measurement Invariance Analysis

Based on the revised two-factor model (two correlated factors with correlations among three general and specific items of positive and negative feelings), this section tests the measurement invariance of the SPANE across gender, age, marital status, education, and income groups. Table 4 summarizes all model fit results from the CFA conducted with Amos 20.0. Due to the large sample size, ΔCFI and ΔMc were used to assess the invariance of different constrained models [28]. According to Cheung and Rensvold (2002), the cutoff values for ΔCFI and ΔMc are −.01 and −.02 [32], respectively.

**Table 4:** Model fit of various invariance models for gender, age, education level and income groups.

Model		df	χ2	RMSEA	SRMR	CFI	ΔCFI	Mc	ΔMc
Gender	Configural invariance	82	4573.9	0.051	0.031	0.975	-	0.899	-
	Weak invariance	92	4678.2	0.048	0.030	0.975	0.000	0.899	0.000
	Strong invariance	95	4694.1	0.048	0.030	0.975	0.000	0.896	−0.003
	Strict invariance	119	5139.4	0.044	0.031	0.972	−0.003	0.891	−0.005
Age	Configural invariance	550	5807.3	0.021	0.034	0.971	-	0.886	-
	Weak invariance	560	5826.8	0.021	0.033	0.971	0.000	0.884	−0.002
	Strong invariance	563	5838.8	0.021	0.033	0.971	0.000	0.883	−0.001
	Strict invariance	587	5940.6	0.021	0.033	0.971	0.000	0.879	−0.005
Marital status	Configural invariance	394	5576.3	0.025	0.0318	0.972	-	0.819	-
	Weak invariance	404	5587.2	0.025	0.0319	0.972	0.000	0.814	−0.005
	Strong invariance	407	5603.3	0.024	0.0327	0.972	0.000	0.804	−0.010
	Strict invariance	431	5873.7	0.024	0.0335	0.970	−0.002	0.785	−0.019
Education	Configural invariance	205	5007.7	0.033	0.053	0.974	-	0.894	-
	Weak invariance	245	5136.7	0.031	0.057	0.973	−0.001	0.889	−0.005
	Strong invariance	257	5370.9	0.031	0.165	0.972	−0.001	0.884	−0.005
	Strict invariance	353	7202.6	0.030	0.211	0.963	−0.009	0.853	−0.031
Income	Configural invariance	205	4997.8	0.033	0.033	0.974	-	0.894	-
	Weak invariance	245	5122.9	0.031	0.035	0.973	−0.001	0.889	−0.005
	Strong invariance	257	5259.1	0.030	0.036	0.972	−0.001	0.891	0.002
	Strict invariance	353	7222.2	0.030	0.045	0.962	−0.010	0.853	−0.038

As shown in Table 4, for measurement invariance across gender, the revised two-factor model adequately fit both male and female data (χ^2^_(82)_ = 4573.9; CFI = .975; SRMR = .031; RMSEA = .051), supporting the configural invariance. Factor loadings were constrained to be equal across male and female groups. This factor-loading-constrained model also fit well (χ^2^_(92)_ = 4678.2; CFI = .975; SRMR = .030; RMSEA = .048). The changed CFI and Mc values between this factor-loading-constrained model and the baseline model were both 0.00, less than the critical values, indicating that there was no difference between the two models and supporting weak invariance. Next, based on the weak invariance model, the variances of factors (structural covariances) were constrained to be equal across gender groups. This further constrained model fit the data well (χ^2^_(95)_ = 4694.1; CFI = .975; SRMR = .030; RMSEA = .048). The values of ΔCFI and ΔMc (ΔCFI = 0; ΔMc = −.003) were both less than the cutoff values, indicating no difference between the further constrained model and the factor-loading-constrained model and supporting strong invariance. Finally, on the basis of strong invariance, uniqueness variance and covariance were constrained to be equal across gender groups. Again, the uniqueness-constrained model fit well (χ^2^_(119)_ = 5139.4; CFI = .972; SRMR = .031; RMSEA = .044). The CFI and Mc difference test indicated that the uniqueness-constrained model was not significantly different from the strong invariance model (ΔCFI = −.003; ΔMc = −.005; both less than the critical values), supporting strict invariance.

The measurement invariance of the SPANE across the eight age groups, six marital statuses, five education levels, and five income levels was examined using the same process. The results in Table 3 indicate that strict equivalence held across different age and marital status groups and that strong equivalence held across different education and income levels.

### Influence of demographic variables on SPANE scores

Independent samples t-tests indicated that females obtained significantly higher scores than males on the SPANE-N scale (t (21320) = 6.91, p<.001), but males obtained significantly higher scores than females on the SPANE-B scale (t (21320) = 4.23, p<.001). No significant difference was found on the SPANE-P scale (t (21320) = .258, p = .796) between males and females. Table 5 presents the correlations between demographic variables and SPANE scores. The point-biserial correlations between gender and SPANE scores are also shown in this table as an index of effect size (females were coded as 0 and males as 1; therefore, a positive correlation indicates higher scores in males).

**Table 5:** Correlations between demographic variables and SPANE scores.

Demographic variables	SPANE-P	SPANE-N	SPANE-B
Gender	.002	−.047**	.029**
Age	.026**	−.039**	.038**
Education level	.045**	−.018**	.036**
Income level	.124**	−.084**	.120**

** , p<.01

### Summary statistics and normative data for the SPANE

Medians, means, standard deviations, ranges, and internal consistency reliability coefficients (Cronbach's alpha) for the SPANE are summarized in Table 6. The Cronbach's alpha coefficients of the SPANE-P, SPANE-N and SPANE-B were all good, with values above .90.

**Table 6:** Summary statistics for the SPANE (*N = 21,322*).

	Median	Mean	SD	Cronbach's alpha	Range
SPANE-P	21	21.03	4.63	0.92	6 to 30
SPANE-N	15	14.37	4.84	0.91	6 to 30
SPANE-B	6	6.66	8.18	0.92	−24 to 24

A Kolmogorov-Smirnov test indicated that none of the SPANE-P (Z = 13.102, p<.001), SPANE-N (Z = 13.890, p<.001) or SPANE-B (Z = 11.786, p<.001) scales were normally distributed. Thus, it is impossible to use the raw score means and SDs from a normative sample to estimate the rarity of an individual's score on these scales [19]. Therefore, norms for the three scales in terms of percentiles are presented in Table 7.

**Table 7:** SPANE scale norms in terms of percentile rankings.

Raw Score	Percentile			Raw Score	Percentile		
	SPANE-P	SPANE-N	SPANE-B		SPANE-P	SPANE-N	SPANE-B
−24	-	-	1	6	1	9	54
−23	-	-	1	7	1	11	58
−22	-	-	1	8	2	14	61
−21	-	-	1	9	2	18	65
−20	-	-	1	10	2	23	69
−19	-	-	1	11	3	27	72
−18	-	-	1	12	5	38	78
−17	-	-	1	13	6	44	80
−16	-	-	1	14	8	50	83
−15	-	-	1	15	10	56	85
−14	-	-	2	16	13	62	87
−13	-	-	2	17	17	68	89
−12	-	-	2	18	33	86	92
−11	-	-	2	19	40	90	93
−10	-	-	3	20	47	93	94
−9	-	-	3	21	53	94	95
−8	-	-	4	22	60	96	96
−7	-	-	4	23	67	97	97
−6	-	-	5	24	82	99	100
−5	-	-	6	25	86	99	-
−4	-	-	7	26	89	99	-
−3	-	-	8	27	91	100	-
−2	-	-	10	28	93	100	-
−1	-	-	13	29	95	100	-
0	-	-	27	30	100	100	-
1	-	-	31				
2	-	-	36				
3	-	-	40				
4	-	-	44				
5	-	-	48				

### Validity: Correlations between the SPANE and Well-Being Measures

To test the validity of the SPANE, correlations among the SPANE-P, SPANE-N, SPANE-B, and two measures of life satisfaction were computed, as shown in Table 8. Support for the convergent validity of the SPANE was provided by large or moderate correlations with measures of well-being. Specifically, the SPANE-P had a large correlation with the *Satisfaction with Life Scale* (r = .68) and the *Satisfaction with Life as a Whole* (r = .56). A similar correlation level was found between the SPANE-B and the SWLS (r = .61) and SWLW (r = .56). Furthermore, the SPANE-N showed moderate correlations with the SWLS (r = −.37) and SWLW (r = −.41).

**Table 8:** Correlations between the SPANE and well-being measures.

	SPANE-P	SPANE-N	SPANE-B
SWLS	.68**	−.37**	.61**
SWLW	.56**	−.41**	.56**
SPANE-N	−.49**	-	
SPANE-B	.86**	−.87**	-

** , p<.01

The correlation between the SPANE-P and SPANE-N subscales was moderate (r = −.49), indicating that they represented related but relatively distinct dimensions of subjective feelings.

## Discussion

Unlike the original research on the development of the new scale using university students [6], the current study used a large sample of full-time employees in China to examine the psychometric properties and dimensionality of the SPANE. The results indicated that the SPANE is a sufficiently reliable and valid measure of subjective feelings of well-being and ill-being. In line with the findings of previous studies [5], [6], the internal consistencies of the overall scale (SPANE-B) and the SPANE-P and SPANE-N subscales were adequate and were all above the cutoff value of .70 [33] or .80 [34]. Furthermore, the convergent validity of the SPANE was supported by revealing moderate to large correlations with two measures of life satisfaction.

Based on the testing of five competing models of the latent structure of the SPANE, the CFA results clearly showed that the correlated two-factor model with the uniqueness of three general and specific items of correlated positive and negative feelings (model 2b) fit the current data best and most adequately. This finding demonstrated that the SPANE-P and SPANE-N scales indexed two distinct but moderately negatively correlated factors when measurement error was controlled. That is, positive and negative feelings were clearly separable although not orthogonal, thus supporting previous conclusions on the separate measurement of positive and negative emotional wellbeing [8], [35] and the hypothesis of Diener et al. (2010) on the development of the SPANE [6]. Silva and Caetano (2013) replicated the two-factor structure of the SPANE by means of principal component analysis and CFA [5]. Unlike the current revised two-factor model, they did not specify the correlations between the uniqueness of three general and specific items of positive and negative feelings. Some authorities on structural equation modeling consider it inappropriate to permit correlated measurement errors for subgroups of items from the same assessment instrument. However, like those in the PANAS as noted by Crawford and Henry (2004) [19], for the SPANE, the correlated errors could be retained because (1) they were specified a priori on the basis of the distinction of the general and specific emotions; (2) with total 12 items, there are 66 potentially correlated errors with only 12 permitted, so the model is far from fully saturated; and (3) the specification of correlated residuals did not substantially change the values of factor loadings or the correlation between the SPANE-P and SPANE-N.

Furthermore, on the basis of the correlated two-factor model, multi-group CFA analyses demonstrated that strict equivalence held across different gender, age and marital status groups and that strong equivalence held across different education and income levels on the SPANE scale. In other words, the factor loadings and correlations between the SPANE-P and SPANE-N do not change with gender, age, marital status, education level, or income level. The error variances and covariances are invariant across different gender, age and marital status groups. The most significant consequence of these results is that that difference in scale scores between subgroups (e.g., the difference between females and males on the SPANE-N scale) can be taken to reflect actual differences in the focus of the construct rather than reflecting artifactual differences in item responses [36]. In particular, the present results are much more meaningful for today's rapidly growing China, where disparities exist between urban and rural areas. Education and household income are two of the most salient indictors [2], [37], [38]. Thus, the current findings provide a solid foundation for an exploration of the differences between Chinese urban and rural areas in terms of emotional well-being.

In response to Diener and his colleagues' (2010) call for the development of norms for groups other than college students [6], we present the SPANE scale norms for employees in terms of percentile rankings. The tabulation method in Table 7 was adopted to permit the transformation of raw scores to percentiles for all three SPANE subscales using the same table. Thus, readers and subjects can determine what the scores signify quickly and easily. For example, if a subject's raw score on the SPANE-P scale was 20, Table 7 indicates that this score is equivalent to the 47^th^ percentile; this score is estimated to be moderate in the employee population. A raw score of 20 on the SPANE-N scale corresponds to the 93^rd^ percentile; this score is rare in the current population. A raw score of 20 on the SPANE-B scale is also rare, with a corresponding 94^th^ percentile.

Some limitations of this study should be addressed in future research. Although the sample size is large and the subjects were from five provinces, all of the subjects were full-time workers from the same industry. Future studies should include broader samples, such as migrant workers, retired people, and farmers in China. Second, because only two life satisfaction scales were included in the convergent validity analysis in the present research, the SPANE scale should be correlated with other measures of subjective and psychological wellbeing, such as the Personal Wellbeing Index and the Basic Needs Satisfaction Scale [39]. Furthermore, the difference and convergence between the SPANE scale and existing emotional well-being scales (e.g., PANAS) should be explored in future studies.

In summary, we achieved the goal of the current research in that our results indicate that the Chinese version of SPANE scale has similar psychometric properties to those demonstrated in two previous studies [5], [6] and showed satisfactory reliability, factorial and convergent validity, and factorial equivalence across demographic variables.
